# Effectiveness and Acceptance of a Smartphone-Based Virtual Agent Screening for Alcohol and Tobacco Problems and Associated Risk Factors During COVID-19 Pandemic in the General Population

**DOI:** 10.3389/fpsyt.2021.693687

**Published:** 2021-07-16

**Authors:** Marc Auriacombe, Lucie Fournet, Lucile Dupuy, Jean-Arthur Micoulaud-Franchi, Etienne de Sevin, Sarah Moriceau, Emmanuelle Baillet, Jean-Marc Alexandre, Fuschia Serre, Pierre Philip

**Affiliations:** ^1^University of Bordeaux, Bordeaux, France; ^2^Sanpsy CNRS USR 3413, Bordeaux, France; ^3^Pôle Interétablissement D'Addictologie, CH Charles Perrens and CHU de Bordeaux, Bordeaux, France

**Keywords:** COVID-19, virtual agent, substance use disorders, technology acceptance, smartphone, eHealth, stress, app intervention

## Abstract

**Background:** During the current COVID-19 pandemic, alcohol, and tobacco are the most available substances for managing stress and can induce a risk of addiction. KANOPEE is a smartphone application available to the general population using an embodied conversational agent (ECA) to screen for experiences of problems with alcohol/tobacco use and to provide follow-up tools for brief intervention.

**Objectives:** This study aimed to determine if the smartphone KANOPEE application could identify people at risk for alcohol and/or tobacco use disorders in the context of the current COVID-19 pandemic, to assess adherence to a 7-day follow-up use diary, and to evaluate trust and acceptance of the application.

**Methods:** The conversational agent, named Jeanne, interviewed participants about perceived problems with the use of alcohol and tobacco since the pandemic and explored risk for tobacco and alcohol use disorder with the five-item Cigarette Dependence Scale (CDS-5) and “Cut Down, Annoyed, Guilty, Eye-opener” (CAGE) questionnaire and experience of craving for each substance. Descriptive, univariate, and multivariate analyses were performed to specify personalized associations with reporting a problem with alcohol/tobacco use; descriptive analysis reported the experience with the intervention and acceptance and trust in the application.

**Results:** From April 22 to October 26, 2020, 1,588 French participants completed the KANOPEE interview, and 318 answered the acceptance and trust scales. Forty-two percent of tobacco users and 27% of alcohol users reported problem use since the pandemic. Positive screening with CDS-5 and CAGE and craving were associated with reported problem use (*p* < 0.0001). Lockdown period influenced alcohol (*p* < 0.0005) but not tobacco use (*p* > 0.05). Eighty-eight percent of users reported that KANOPEE was easy to use, and 82% found Jeanne to be trustworthy and credible.

**Conclusion:** KANOPEE was able to screen for risk factors for substance use disorder (SUD) and was acceptable to users. Reporting craving and being at risk for SUD seem to be early markers to be identified. Alcohol problem use seems to be more reliant on contextual conditions such as confinement. This method is able to offer acceptable, brief, and early intervention with minimal delay for vulnerable people.

## Introduction

Since December 2019, cases of severe acute respiratory syndromes due to Coronavirus 2 (SARS-CoV-2) also named COVID-19 (1) were reported in Wuhan, China, and have spread around the world within months. The ease of transmission of COVID-19 in the general population, the incidence of severe complications requesting access to intensive care medical facilities, and the associated mortality and lack of specific treatment led health authorities in most countries to isolate their population in lockdown quarantine procedures (2). Such crisis management procedures including isolation, social distancing, maintained confinement, wearing of masks, and cancellation of family, social, and cultural events in addition to individual health concerns are all potential stressors (3, 4). It is suggested that the COVID-19 negatively impacts mental health beyond health-related fears (5) and induces negative psychological effects (3, 4, 6–8). COVID-19 could also negatively impact mental health by a direct action of the virus in the brain (5). Hence, COVID-19 could intensify use of addictive substances as coping or stress relief strategies (9, 10).

Tobacco and alcohol are the most accessible substances worldwide (7) and remain readily accessible as they are included in “essential purchases” such as food in most countries (11). Use of these substances has increased during the lockdown (12). Few studies have directly monitored alcohol and tobacco use in the context of past lockdowns. In their review on the mental health impact of lockdown, Brooks et al. (3) recently reported only one study involving Chinese healthcare workers mobilized during the SARS epidemic in 2003. In this study, quarantine was the main predictor of alcohol addiction 3 years later. Among alcohol users, those who drank with the intention of “coping with the epidemic” had six times more symptoms of alcohol abuse and dependence than those who did not drink to cope. Also, the symptoms of addiction persisted 3 years later (6).

Recently, concern was expressed about the increase in tobacco use and relapse among smokers due to the decrease or absence of usual sources of regulation (5). In people diagnosed with addiction, negative emotions (e.g., symptoms of mood or anxiety disorders) are reported to be among the factors that increase substance use and relapse risk, even in long-term abstainers (7, 13, 14). In the general population, diverse increases in addictive behaviors were found during lockdown and have been linked with decreased well-being and increased stress, considered as universal risk factors (15–17). When other adaptive means of managing stress are absent (e.g., socializing and keeping one's mind occupied), craving may increase (8).

During the development of an addiction, use is no longer contextual and can continue outside of the stressful period in which it began. Independent of these environmental factors, individual factors contribute to alcohol and tobacco use. These include use disorders, mental disorders, and individual responses to stress (18). To reduce alcohol and tobacco use, it is sometimes possible to act on environmental stress factors; however, in the event of an epidemic or natural disaster, it is not possible. In such cases, it is important to act on individual factors through information and specific identification of at-risk individuals for personalized focused interventions. Research suggests that craving could be among the earliest diagnostic markers of addiction, potentially prior to the occurrence of addiction-induced damages (19). Craving is suggested to be a prognostic marker of substance use disorders (SUDs) (13, 20, 21). Consequently, interventions should focus more on individuals at-risk of developing an addiction instead of addressing the general population globally. The goal would be to act on risk factors for use disorder.

The current COVID-19 crisis requires new means for identification of health status, while maintaining the principles of reducing person-to-person contact and contamination. In this context, a proactive approach to monitoring alcohol and tobacco use in the general population is essential. Protocols may be oriented toward the development of virtual and/or distant resources and the reduction of face-to-face visits (6). During the first lockdown in 2020, numerous research projects have emerged to assess the experiences of individuals and the evaluation of various mental disorders in the most ecological way possible (22). It is in this context that KANOPEE was developed.

KANOPEE is the first application of embodied conversational agents (ECAs), otherwise named virtual agents, whose intended purpose is to detect and prevent problems with tobacco and alcohol use as well as sleep disorders related to the COVID-19 pandemic (23–25). It combines a weekly assessment by the virtual agent and a daily monitoring of substance use by an electronic diary, which have been shown to be effective on reducing alcohol and tobacco intake (26, 27). The virtual agent used in KANOPEE for alcohol and tobacco use disorder screening has already shown good acceptance, validity, and reliability in a hospital setting (23, 25) but has never been deployed in the general population. Results of the feasibility of KANOPEE regarding screening and intervention for insomnia symptoms have been presented elsewhere (28).

KANOPEE was launched during the COVID-19 pandemic to respond to the immediate needs of addressing the mental health of the French population in that context. In this first report on the use of the KANOPEE smartphone application for alcohol and tobacco assessment, our objectives were to identify people at risk for alcohol and/or tobacco use disorders and to determine if the application could determine personalized risk factors associated with reporting problems with alcohol/tobacco use. This was operationalized as examining if self report of problems with alcohol/tobacco use during current COVID-19 pandemic were associated with being at risk of SUD, reporting tobacco/alcohol craving, being a COVID-19 healthcare worker, and being confined due to pandemic lockdown restrictions. Moreover, characteristics of the follow-up respondents were studied to examine the profile of users of the diary. Lastly, trust toward the virtual agent and acceptance of the overall system were measured, and we analyzed if user characteristics influenced attitude toward the virtual agent.

## Materials and Methods

### Participants and Recruitment

**Figure 1:**
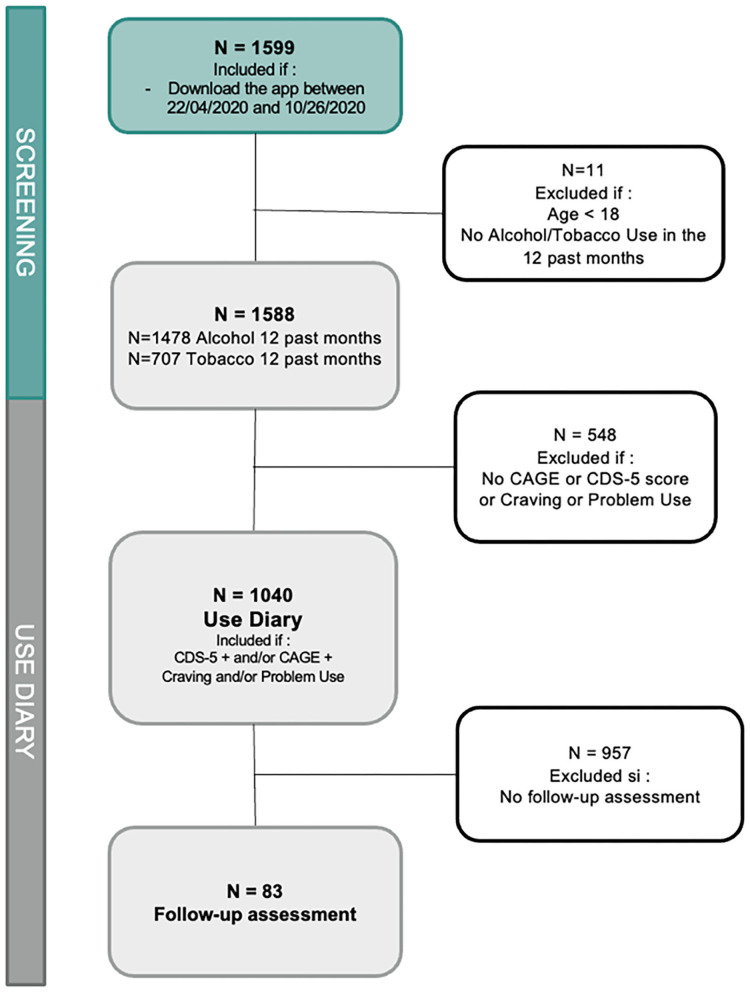
Flowchart of users included in the different steps of analyses.

Data were collected from users of the KANOPEE application, downloaded voluntarily between April 22 and October 26, 2020. Respondents were anonymous and had to meet the following inclusion criteria: be of legal age (18 years old and over), have used tobacco or alcohol at least once in the past 12 months, and have completed the screening interview for tobacco and alcohol use disorder [five-item Cigarette Dependence Scale (CDS-5) and/or “Cut Down, Annoyed, Guilty, Eye-opener” (CAGE) questionnaire]. Figure 1 summarizes the recruitment procedure.

### Ethics

The approval of the scientific committees of the University of Bordeaux was obtained as well as the agreement with respect to the General Data Protection Regulations (GDPR) by the French authorities (CNIL). Informed consent was obtained directly on the app by all participants who downloaded the application.

### Description of KANOPEE Implementation and Functionalities

KANOPEE is a smartphone application developed by the University of Bordeaux (SANPSY CNRS USR 3413) in partnership with CHU Bordeaux and CH Charles Perrens. It is available for Android and Apple IOS and for free download since April 2020. It provides weekly interactions with a virtual agent named Jeanne, proposing a screening evaluation assessing lifetime and current (past year) alcohol and tobacco use, craving for alcohol and tobacco, and risk of SUD. Furthermore, Jeanne inquires about experiencing problems with alcohol or tobacco use since the COVID-19 epidemic.

For users identified as “at risk” (CDS-5 > 9 or CAGE > 1, reporting craving, and reporting of alcohol/tobacco problem use due to the epidemic), a daily use diary was proposed.

The diary was designed to help the user to monitor craving and daily alcohol and tobacco intake and to confront it by self-determined daily use objectives. The diary was initially available as a downloadable printable version and then was integrated within the application in an update (May 21, 2020). After 7 days and then every 7 days thereafter, a follow-up evaluation with Jeanne asked participants on whether the application and diary were helpful. After 2 weeks of use, the application questioned participants on whether direct contact with a healthcare worker was required.

### Measures

Variables collected by KANOPEE and extracted for this study were as follows:

- *Age, gender, level of education*, and *socio-professional category (SPC)* collected as sociodemographic data.
- *Period of confinement*: according to the date of the initial KANOPEE interview. In France, the period of confinement was from April 22, 2020, to June 1, 2020, and the post-confinement period was from June 2, 2020, onward.
- *COVID-19 healthcare worker*: provided by answering yes to the question “Are you currently involved in the care of patients infected with COVID-19?”
- *Reported problem with alcohol/tobacco use during the epidemic*: provided by answering yes to the question “In the context of the COVID-19 epidemic, did you feel that your situation with respect to tobacco/alcohol has worsened?”
- *Risk of SUD*: Screening questionnaires were previously adapted to be performed by an ECA (23, 25). The scores were dichotomized (positive/negative) according to their respective threshold scores. The CDS-5 evaluates tobacco dependence and has been validated and considered as a reliable measure (29). Scores vary from 5 to 25, and the threshold score for dependence was set at 9 in accordance with the DSM-5 (23). “Cut Down, Annoyed, Guilty, Eye-opener” evaluates problem drinking (30, 31). The embodied conversation agent version of the CAGE validation study showed that a score >1 detected a problem with alcohol, with scores varying from 0 to 4 in accordance with DSM-IV and DSM-5 (23).
- *Craving*: determined by answering yes to “Could you tell me if, in the past 12 months, you have felt a persistent desire, a very strong craving, or an irresistible need to smoke tobacco or cigarettes/drink alcohol?”
- *Helpful perception of the diary*: For users of the diary, the following question was asked in the weekly follow-up evaluation: “Did last week's advices and use of the diary help you?” (scale of 1 to 7, scores were dichotomized from 1 to 3 = “not helped” and from 4 to 7 = “helped”).
- *Acceptance and trust questionnaires*: After the interviews with the virtual agent, users could complete two assessments on the application. The French version of the Acceptability E-scale (16, 32) measured acceptance of the KANOPEE application based on two subscores: usability (the perceived ease of using the system or application) and satisfaction (the perceived enjoyment of the use and usefulness of the system or application). The ECA trust questionnaire (ETQ) (25) measured a user's trust in a virtual agent based on two sub-dimensions: perceived credibility (perception that the agent has the ability and the expertise to conduct a medical intervention) and benevolence (perception that the agent is well-intentioned and will accurately take the user's interests into account).
- *Familiarity with technologies*: evaluated by the single question “Are you familiar with computer technologies?” with the following three choices: “No,” “Moderately,” and “Yes,” which were scored as 0, 1, and 2, respectively.

### Statistical Analyses

We described continuous variables by means (*M*) and standard deviations (*SD*) and categorical variables by frequencies and percentages (%). The dependent variable was the *reported problem with alcohol/tobacco use due to the epidemic*, and explanatory variables were to present a *risk of SUD, craving*, using the application during the *period of confinement*, and being a *COVID-19 Healthcare worker*. Analyses were carried out separately for tobacco and alcohol users. Descriptive and univariate analyses were first conducted.

To determine risk factors for *reporting a problem with alcohol/tobacco use due to the epidemic*, we performed chi-square analyses for these categorical explanatory variables. Multivariate logistic regression analyses were conducted to test these associations found in univariate analysis, controlling for potential confounders (*period of confinement, age, gender, level of education*, and *SPC*). The proportion of the explained variance of multivariate models was examined with the *R*^2^(*U*) value. Univariates analyses were also conducted to identify variables associated with completing follow-up assessments and finding the application and diary *helpful*. Lastly, to investigate factors associated with acceptance and trust, we conducted univariate analyses with Pearson correlation analyses between two continuous variables (*age, risk of SUD*, and *familiarity with technologies*) and performed mean comparisons (*t*-test or analysis of variance) to analyze the variation in AES and ETQ scores regarding categorical variables (gender and educational level).

Analyses were performed using the JMP Pro 14® software. The significance threshold was set at *p* ≤ 0.05.

## Results

### Sample Description

From April 22, 2020, to October 26, 2020, 1,588 subjects downloaded the application and answered at least one interview on tobacco and/or alcohol from the KANOPEE application (Table 1).

**Table 1:** Socio-demographic, use, confinement, and evaluation characteristics of users of the KANOPEE application.

	**KANOPEE (*N* = 1,588)**		**Follow-up (*N* = 83)**			**Helped (*N* = 38)**		
	***N***	**% or *M* (SD)**	***N***	**% or *M* (SD)**	***p*****-value**	***N***	**% or *M* (SD)**	***p*****-value**
Age (years)	1,588	43.56 (13.67)	83	43.96 (1.50)	0.7827	38	44.32 (2.30)	0.8270
Gender (women)	898	56.55	48	57.83	0.8085	20	52.63	0.3780
COVID-19 healthcare worker	127	8.00	12	14.46	**0.0420**	8	21.05	0.1154
Period of confinement	1,063	66.44	46	55.42	**0.0250**	19	50	0.3610
**Level of education**					0.3918			0.5477
French certificate of general education	259	16.31	17	20.48		10	26.32	
Baccalaureate	286	18.01	10	12.05		5	13.16	
Bac. to Bac.+5	840	52.90	44	53.01		19	50	
More than Bac.+5	203	12.78	12	14.46		4	10.53	
**Socio-professional category (SPC)**				0.4161			0.7883
Farmers	29	1.83	0	0		0	0	
Entrepreneurs and self-employed	81	5.10	4	4.82		1	2.63	
Higher managerial and professional occupations	482	30.35	23	27.71		9	23.68	
Intermediate occupations	127	8.00	6	7.23		3	7.89	
Employees, tertiary	486	30.60	30	36.14		16	42.11	
Trade and factory workers	61	3.84	1	1.20		0	0	
Retired	160	10.08	8	9.64		4	10.53	
Inactive	162	10.20	11	13.35		5	13.16	
**Substances**								
**Tobacco**								
Lifetime past use of tobacco	1,372	86.40						
Past-year use of tobacco	708	51.60						
CDS-5 Score > 9	496	70.06	28	33.73	0.7881	11	28.95	0.5355
CDS-5 Mean Score		13.34 (5.69)						
Craving	592	83.62	37	44.58	0.1934	15	39.47	0.0777
Reported tobacco problem use	297	41.95	23	57.50	**0.0434**	7	38.89	**0.0300**
**Alcohol**								
Lifetime past use of alcohol	1,550	97.61						
Past-year use of alcohol	1,478	95.35						
CAGE > 1	482	32.61	32	38.55	**0.0175**	13	34.21	0.3816
CAGE Mean Score		0.95 (1.67)						
Craving	416	28.15	32	38.55	**0.0034**	16	42.11	0.4684
Reported alcohol problem use	413	27.94	30	36.59	0.0809	13	34.21	0.6779
**Follow-up evaluation (***N*** = 83)**								
Help	38	45.78						

*Bold values mean that their p is significant (<0.05)*.

The sample mean age was 44 years old (*M* = 43.56, *SD* = 13.67), mostly women (*N* = 898, 56.54%), and half had a high level of education (≥12 years) (*N* = 840, 52.90%). Two-thirds (*N* = 1,063, 66.44%) responded during the confinement period (from April 22 to June 1, 2020). One hundred twenty-seven (8%) subjects reported being COVID-19 healthcare workers.

Regarding tobacco and alcohol use, half of the sample (*N* = 708, 51.60%) reported tobacco use during the past year, and among them, 83.85% (*N* = 592) reported craving for tobacco. The mean CDS-5 score was 13.34 (*SD* = 5.69), and 496 (70.16%) tobacco users were at risk of tobacco use disorder (CDS-5 score > 9). Almost the entire sample reported past-year alcohol use (*N* = 1,478, 95.29%), and among them, 28.17% (*N* = 416) reported craving for alcohol. The mean CAGE score was 0.95 (*SD* = 1.67), and 482 (32.61%) alcohol users were at risk of alcohol use disorder (CAGE score > 1).

Six hundred and fifty-five subjects (41.25%) used both substances, and 11% (*N* = 177) were at risk for both alcohol and tobacco use disorder (CDS-5 score > 9 and CAGE > 1).

### Reported Problem With Alcohol/Tobacco Use and Associated Factors

A higher proportion of current tobacco users (41.95%, *N* = 297) than that of alcohol users (27.94%, *N* = 413) reported a problem use since the context of the epidemic.

Results of univariate analyses (Table 2) suggest that for both alcohol and tobacco users, *risk of SUD diagnosis* (for the substance concerned) was associated with more frequent reports of problem with alcohol/tobacco use (*p* < 0.0001). *Being a COVID-19 healthcare worker* increased the risk of reporting a problem with alcohol use during the current epidemic (*p* = 0.0463) but did not increase the risk to report a problem with tobacco use. There was no significant difference between the proportion of those who reported a problem with alcohol/tobacco use according to period of use of the application (during the confinement period or after) (*p* > 0.05 for tobacco and alcohol).

**Table 2:** Factors associated with reported tobacco and alcohol problem use in the context of the COVID-19 pandemic (univariate analyses).

**Characteristics**	**TOBACCO (*N* = 708)**		**ALCOHOL (*N* = 1,478)**	
	**Reported problem use**	***p*****-value**	**Reported problem use**	***p*****-value**
**Age**	*N* (%)		*N* (%)	
18–45	213 (44.83)	0.2363	251 (30.80)	**0.0009**
45–65	81 (38.94)		146 (26.50)	
>65	3 (25.00)		16 (14.41)	
**Gender**				
Women	197 (45.81)	**0.0119**	238 (29.02)	0.3095
Men	100 (36.23)		175 (26.64)	
**Level of Education**				
French Certificate of General Education	55 (41.98)	0.3089	53 (23.87)	0.1312
Baccalaureate	59 (39.86)		68 (25.76)	
Bac. to Bac.+5	164 (44.57)		242 (30.48)	
More than Bac.+5	19 (32.20)		50 (25.38)	
**SPC**				
Farmers	8 (66.67)	0.0666	9 (33.33)	**0.0010**
Entrepreneurs and self-employed	12 (26.67)		23 (29.49)	
Higher managerial and professional occupations	78 (44.07)		143 (30.69)	
Intermediate occupations	27 (49.09)		47 (38.52)	
Employees, tertiary	123 (47.13)		125 (27.84)	
Trade and factory workers	8 (33.33)		9 (18.00)	
Retired	5 (22.73)		22 (14.97)	
Inactive	36 (32.73)		35 (25.36)	
**Risk of SUD**				
CDS-5 > 9 or CAGE > 1	251 (50.60)	**<0.0001**	234 (50.41)	**<0.0001**
CDS-5 ≤ 9 or CAGE ≤ 1	46 (21.90)		170 (17.09)	
**Craving**				
Craving	281 (47.47)	**<0.0001**	208 (50.00)	**<0.0001**
No craving	16 (14.04)		205 (19.32)	
**COVID-19 Healthcare Worker**				
Healthcare worker	24 (40.00)	0.7344	42 (35.90)	**0.0463**
Not a healthcare worker	273 (42.26)		371 (27.28)	
**Period of Confinement**				
Confinement	198 (43.90)	0.1892	294 (29.43)	0.0693
Post-confinement	99 (38.82)		119 (24.90)	

*Bold values mean that their p is significant (<0.05)*.

Multivariate analysis (Table 3) showed that for tobacco, reporting problem use during the pandemic was associated with being at risk of tobacco use disorder (aOR = 2.7264, CI = [1.78–4.18], *p* < 0.0001) and experiencing craving (aOR = 3.3608, CI = [1.80–6.27], *p* < 0.0001).

**Table 3:** Factors associated with reported tobacco and alcohol problem use in the context of the COVID-19 pandemic [multivariate analyses with odds ratio (OR)].

	**TOBACCO**			**ALCOHOL**		
***Reported problem use***	***aOR***	***CI 95%***	***p-value***	***aOR***	***CI 95%***	***p-value***
**Age**			0.3451			0.3501
(18–44]	1.1660	0.13–5.48	0.8711	1.7751	0.80–3.95	0.1602
(45–65]	0.8882	0.14–5.53	0.8989	1.6185	0.75–3.51	0.2223
**Gender**			0.1298			0.0849
(Men]	0.7665	0.54–1.08		0.7904	0.60–1.03	
**Level of education**			0.6136			0.7146
(Baccalaureate]	0.8498	0.50–1.43	0.5421	0.9065	0.57–1.44	0.6757
(Bac to Bac.+5]	1.0526	0.66–1.68	0.8310	0.7356	0.42–1.27	0.2732
(More than Bac.+5]	0.7450	0.50–1.43	0.5421	0.9163	0.57–1.44	0.2814
**SPC**			**0.0018**			**0.0414**
(Farmers]	9.3105	1.33–65.42	**0.0249**	2.1868	0.75–6.35	0.1505
(Trade and factory workers]	1.2559	0.23–6.75	0.7906	0.7958	0.29–2.20	0.6596
(Employees, tertiary]	2.2256	0.52–9.44	0.2779	1.3712	0.67–2.79	0.3843
(Inactive]	1.1041	0.25–4.89	0.8962	1.0679	0.48–2.38	0.8724
(Entrepreneurs and self-employed]	1.1774	0.23–5.43	0.8906	1.7768	0.75–4.18	0.1882
(Higher managerial and professional occupations]	2.6653	0.61–11.56	0.1903	1.7168	0.85–3.49	0.13.51
(Intermediates occupations]	3.0336	0.65–14.09	0.1340	2.5542	1.17–5.60	**0.0192**
**COVID-19 Healthcare Worker**			0.9444			0.1772
(No]	0.9785	0.53–1.18		0.7262	0.46–1.15	
**Risk of SUD**			**<0.0001**			**<0.0001**
(Yes]	2.7442	1.79–4.12		3.7979	2.88–5.02	
**Period of confinement**			0.3133			**<0.0001**
(No]	0.8381	0.59–1.18	0.3139	0.5742	0.43–0.76	
**Craving**			**<0.0001**			**<0.0001**
(Yes]	3.3719	1.80–6.28		2.6659	2.01–3.53	

***Reference levels**: Age = more than 65 years old; gender = women; level of education = French Certificate of General Education; SPC = retired; COVID-19 healthcare workers = Yes; risk of SUD = no; period of confinement = yes; craving = no*.

*Ordinal logistic regression models. Reported tobacco problem use: n = 708; R^2^(U) =0.1074; χ^2^ = 103.17; dof = 17; p <0.0001. Reported alcohol problem use: n = 1,478; R^2^(U) = 0.1511; χ^2^ = 264.48; dof = 17; p <0.0001*.

*Bold values mean that their p is significant (<0.05)*.

For alcohol, reporting problem use was associated with being at risk of alcohol use disorder (aOR = 3.8002, CI = [2.88–5.02], *p* < 0.0001), experiencing craving (aOR = 2.6481, CI = [1.99–3.51], *p* < 0.0001), and being currently in a period of confinement (aOR = 0.5904, CI = [0.44–0.79], *p* < 0.0005).

The whole model explained approximately 11% of the variations of reporting tobacco problem use [*R*^2^(*U*) = 0.1074] and around 15% of reporting alcohol problem use [*R*^2^(*U*) = 0.1511].

### Follow-Up Assessment and Perceived Help From the Diary

Of the 83 respondents to the 7-day follow-up evaluation, 45.59% (*N* = 38) found the application and the diary helpful (Table 1).

There was no difference in terms of age, gender, socio-professional categories, and level of education between the initial and follow-up samples (*p* > 0.05 for all).

COVID-19 healthcare workers were more likely to fill the 7-day follow-up evaluation (*p* < 0.05) and to complete it outside of the confinement period (*p* < 0.05). For alcohol, subjects who filled the 7-day evaluation were more likely to report a use disorder risk (*p* = 0.0175) and craving for alcohol (*p* = 0.0034). For tobacco, subjects who filled the 7-day evaluation were more likely to report tobacco problem use (*p* = 0.0434).

Compared to the initial sample, reporting that the application and diary were helpful was more frequent for those who reported a tobacco problem use (*p* = 0.0300).

### Trust and Acceptance of KANOPEE

**Figure 2:**
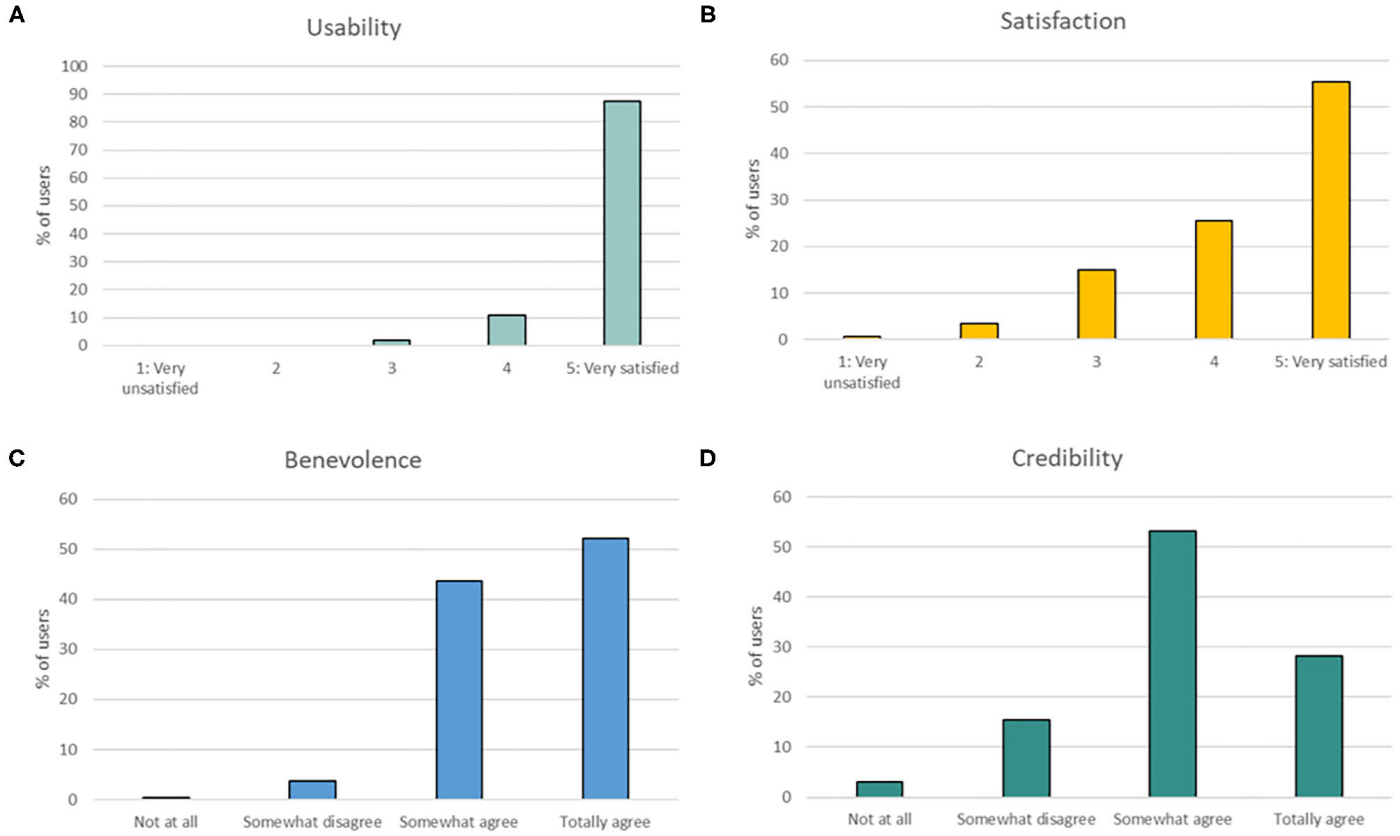
Perception of usability, satisfaction of KANOPEE, and benevolence and credibility of the virtual agent (Jeanne). **(A)** Percentage of users' rating for the usability dimension (AES subscore), **(B)** percentage of users' rating for the satisfaction dimension (AES subscore), **(C)** percentage of users' rating for the benevolence dimension (ETQ subscore), and **(D)** percentage of users' rating for the credibility dimension (ETQ subscore).

Overall, 318 of 1,588 (20.0%) users answered the acceptance and trust questionnaires (Figure 2).

Acceptance of the overall system (AES score) was rated very positively, with 87.4% (278/318) of users being “very satisfied” with the usability of the system, and 80.0% (267/318) of users rating the virtual agent more than 3 out of 5 for satisfaction. Regarding trust (ETQ score), Jeanne was perceived as trustworthy to perform medical interviews. Indeed, 95.9% (305/318) of users “somewhat agreed” or “totally agreed” that she was benevolent, and 81.44% (259/318) of users had a positive attitude toward her credibility (i.e., rating of more than 1 out of 3).

Interestingly, correlation analyses revealed that both subscores of ETQ were positively correlated with both subscores of AES, with high coefficients of correlation, suggesting that the more Jeanne was perceived credible and benevolent, the more the whole application was found usable and satisfactory (*r* = 0.564; *p* < 0.001).

Regarding the influence of user characteristics, correlation analyses revealed a positive relation between the usability subscore of AES and education level, suggesting that users with a higher education level found KANOPEE more usable (*r* = 0.121; *p* = 0.031). Similarly, users with a higher familiarity with technologies found KANOPEE more usable (*r* = 0.156; *p* = 0.003).

Other user characteristics (i.e., age, gender, education level, being a healthcare worker, and risk of SUD) did not reach a significant level of correlation between any of the subscores of AES and ETQ, suggesting that the attitude toward Jeanne and the application was not influenced by these factors.

## Discussion

The KANOPEE smartphone application was designed to address the general population's mental health needs in the context of the current pandemic. In this study, we focused on the application's ability to access those at risk to report problem with alcohol/tobacco use, in view of facilitating early access to personalized prevention. In this report of an initial sample of 1,588 participants covering the first French general population confinement, 42% of tobacco users (297/708) and 28% of alcohol users (413/1,478) reported problem use due to the pandemic context. Self-report of craving and screening for SUD diagnosis by the KANOPEE application were the most consistent associated risk factors to report problem with alcohol/tobacco use. The application's self-help daily diary was used by those with increased risk, and overall, 46% reported it was helpful.

Alcohol use was reported by almost the whole sample, while a little less than half used tobacco in the past 12 months. Compared to the French general population, our sample had more smokers. In 2019, 30.4% of people aged 18–75 years reported smoking tobacco (33) compared to 44.58% in our sample. For alcohol, 30.35% of our sample were identified with risky use of alcohol, which is higher than what was found (8.6% to 23.6%) in a general population study aged 18–75 years (34). Thus, it is possible that the application was more appealing for at-risk individuals (22).

Current tobacco users were more frequently identified as being at risk of an SUD diagnosis (around 70% with CDS-5) than alcohol users (around 33% with CAGE), and they were more likely to report a problem of use since the context of the epidemic. It is known that smoking tobacco is more addictive than drinking alcohol (35). Thus, even if alcohol is largely used in the general population, tobacco use confers a greater potential to report a problem use in the context of the pandemic. This may be due to the higher addictive potential of tobacco compared to alcohol.

Reporting a problem with alcohol use since the epidemic was more frequent among those who completed the application during the period of the first lockdown in France, which was not the case for tobacco users. Alcohol use may be more influenced by immediate contextual variations than tobacco use. Our results suggest that the KANOPEE application was able to show that if smoking may not be influenced by periods of confinement, in opposition to drinking alcohol, the overall impact of the epidemic was more important for tobacco users. Previous studies reported no or little change in tobacco use or request for support since the beginning of COVID-19 while one-half of alcohol users reported a change in alcohol use (36, 37), and sometimes up to 30% reported an increase in alcohol use (12). In a Belgian study, even if alcohol and tobacco increases were related to COVID contextual variables (e.g., work at home and unemployment), reasons for use were different (i.e., sociability for alcohol and boredom for tobacco). These results lead us to pay more attention to specificities across substances (15, 16) and notably motivation for use. Furthermore, between 11 and 15% of reported problem use was explained by the studied variables. This result testifies that, even if variables known to be associated with problem use play a role in their occurrence during the epidemic, further studies should explore other factors in understanding the development of use-related problems in this context.

On another note, characteristics associated with the use of the diary were to be a COVID-19 healthcare worker, using the application out of the confinement period, reporting a tobacco problem use, being at risk of alcohol/tobacco use disorder, and reporting craving for alcohol. Ultimately, the only characteristic related to being most helped by the application was reporting a problem with tobacco use. Thus, even if the addictive core (being at risk of use disorder and presence of craving) is associated with follow-up completion, it is the subjective report of a problem use that is associated with the feeling of being helped by the KANOPEE application. This result is therefore promising for the use of KANOPEE by tobacco users (the most prevalent addiction), and further studies could explore why problems with tobacco use is associated with helpfulness of the diary.

In this study, craving appeared to represent a marker of risk for reporting a problem with alcohol/tobacco use as much as screening with CDS-5 and CAGE. Research exploring the different SUD diagnostic criteria has reported craving as among the most consistently frequent and discriminant criteria across different SUDs, including alcohol and tobacco (38, 39). Thus, craving could be the focal point of personalized early identification and the main objective of prevention of alcohol/tobacco problem use in the context of the pandemic.

Lastly, regarding acceptance and trust measures, we reproduced in the general population previous results observed in a hospital setting (25), suggesting that trust and credibility of a virtual agent are crucial to promote acceptance of a healthcare digital solution. We noted that user characteristics had only a limited influence on trust and acceptance of the application, suggesting that KANOPEE was appreciated by all, regardless of user's condition.

Some limitations must be acknowledged. First, this study was conducted in a sample recruited through a mobile application and not through face-to-face data collection. As a result, it is possible that some of the data could be erroneous (e.g., voluntarily false answers and multiple registrations by same individual). Nevertheless, previous studies from general-population internet-based surveys have shown that the data generated may be reliable (40). Furthermore, our data are consistent with current knowledge. Second, the application did not collect information on quantity or frequency of tobacco and alcohol use before the epidemic for objective comparison with current use. Participants were not directly questioned about changes in quantities of alcohol and tobacco use since the COVID-19 pandemic but were asked whether their situation with tobacco or alcohol use had worsened since the beginning of the epidemic. This question offered access to participants with sufficient insight to perceive having a problem with use of tobacco and alcohol and the capacity to report it. If it is unlikely that those reporting problem use would not report an increase in quantity and/or frequency of use if measured directly, we cannot exclude however that among those not reporting a problem use, some may have increased quantity and/or frequency of use. Furthermore, reporting problem use could not be due to a lack of access and unvoluntary reduced use, since tobacco and alcohol remained readily available throughout the different phases of the epidemic, including confinement periods. Also, the use diary was helpful for almost half of the users but used by a minority only. Low ongoing use has been found in other populations using a mental health application during COVID (22). In general, the use of mental health applications is waning over time, and a small proportion of users actually use popular applications regularly (41). Finally, no psychiatric comorbidity was assessed, being out of the scope of our addiction-focused application. Our main objective was to propose an early identification of addiction risk in the general population regardless of psychiatric comorbidity. In order to further increase adherence, appearance and user-friendliness of the diary could be improved (27, 42).

## Conclusion

Overall, for the first time, our data suggest that the KANOPEE application was able to access, within the French general population, a group at risk of experiencing problems with alcohol and tobacco use in the context of the COVID-19 epidemic. Our results are consistent with other reports that the epidemic has increased alcohol and tobacco misuse for some users, and in addition, we were able to characterize potential risk markers for prevention and early intervention: reporting craving and screening for risk of SUD. The promotion of KANOPEE takes place in the context of a growing demand for the use of digital technology (e.g., remote assessments and teleconsultations) in the context of the pandemic. More research is needed to confirm the markers for risk of experiencing problem use with alcohol and tobacco and to determine if the early monitoring with the diary is effective as an early intervention.

## Data Availability Statement

The raw data supporting the conclusions of this article will be made available by the authors, without undue reservation.

## Ethics Statement

Ethical review and approval was not required for the study on human participants in accordance with the local legislation and institutional requirements. The patients/participants provided their digital informed consent to participate in this study.

## Author Contributions

MA was the PI for the application of KANOPEE to substance use and addiction and obtained funding. PP was the overall PI of the KANOPEE project and obtained funding. LF, FS, and LD analyzed the data. LF and MA drafted the initial manuscript. LD, FS, EB, J-MA, and J-AM-F provided methodological support, critical revision, and editing of the manuscript. ES provided technical support on data and application. All authors significantly contributed to the manuscript and approved the final version.

## Conflict of Interest

The authors declare that the research was conducted in the absence of any commercial or financial relationships that could be construed as a potential conflict of interest.
